# An intelligent incentive-based demand response program for exhaustive environment constrained techno-economic analysis of microgrid system

**DOI:** 10.1038/s41598-025-85175-z

**Published:** 2025-01-06

**Authors:** Bishwajit Dey, Gulshan Sharma, Pitshou N. Bokoro, Soham Dutta

**Affiliations:** 1 Department of Electrical Engineering Technology, University of Johannesburg, Johannesburg, 2006 South Africa; 2 Department of Electrical and Electronics Engineering, Manipal Institute of Technology, Manipal Academy of Higher Education, Manipal, India

**Keywords:** Microgrid, Incentive-based demand response, Energy management, Electricity market price, Optimization, Engineering, Electrical and electronic engineering, Energy infrastructure

## Abstract

The cost-effective scheduling of distributed energy resources through sophisticated optimization algorithms is the main focus of recent work on microgrid energy management. In order to improve load factor and efficiency, load-shifting techniques are frequently used in conjunction with additional complex constraints such as PHEV scheduling and battery life assessment. Pollutant reduction, however, is rarely highlighted as a primary goal. An incentive-based demand response (IBDR) is introduced in the proposed work to close this gap and promote load curtailment during peak hours. IBDR policy rewards participant customers with incentives for load curtailment which in turn lowers emissions and generation costs. Furthermore, a trade-off approach ensures both environmental and economic sustainability by striking a balance between cost reduction and emission reduction. Considering the fact in view that the 30–40% of the microgrid customers are willing to participate in the IBDR program, six different scenarios that have been analysed, each of which involves various levels of grid participation and different approaches to pricing in the electricity market. These scenarios also include the implementation of demand response programmes. Differential evolution algorithm was used as the optimization tool for the study. The results achieved for all the scenarios demonstrate the suitability and effectiveness of implementing the suggested IBDR strategy in terms of cost savings. According to numerical results reported, the generating cost decreased by 10–13% with the inclusion of IBDR. Additionally, a 6–8% reduction in peak and 4–5% improvement in load factor was also realised as a positive impact of the IBDR policy. The weighted economic emission dispatch algorithm offered a balanced solution that considered both the minimum generation cost and emissions for various load models in the microgrid system.

## Introduction

Renewable energy has made its way into the realm of ELD, where its principles are being increasingly implemented. The perception of ELD replicates that all the components associated do not cater to the same expanse of load as per the same amount of cost, i.e., the cost per unit of production will vary for different units. However, the proper operation of any power grid imposes the equal balance between the entire energy production and the entire energy demand^1^. Based on the load demand, ELD can be divided into two categories: ScELD and DcELD. In certain situations, the load demand remains constant for a set period, which is known as ScELD. On the other hand, DcELD refers to scenarios where the load demand fluctuates within a specific interval. Due to the relatively less limited number of restraints, ScELD is simpler^2,3^ whereas DcELD has a more difficult optimization problem. Managing the various constraints related to DERs, time periods, and ScELD^4^ can be quite challenging. The most crucial aspect of achieving the best solution for DcELD problems is determining the start and end times of DERs, particularly when storage devices are in use.

### Related work

An in-depth analysis of ELD can be seen as a challenging subject in the electrical power industry. In situations involving non-convex, continuous, and smooth cost functions, the typical computational methods can be readily implemented^5^. However, when the confirmed physical limits are taken into account, the ELD problem becomes more complex and challenging due to its non-smooth and convex nature. This complexity makes the usual approaches ineffective^6^. To address the smooth ELD issues, common optimisation techniques such as the gradient method, dynamic programming, and iterative lambda are often employed^7^. However, accurately modelling ELD problems in real scenarios necessitates a high level of precision and careful management of various constraints. This results in a more intricate objective function that challenges the established strategies for achieving the best possible solution^8^. The nature of the cost curve poses one of the most interferences to the usage of these methods. Dynamic programming is unaffected by the cost curve, although system dimensionality has a big effect on its management. In addition, the dynamic programming algorithm can be computationally intensive when dealing with large systems^9^.

With the use of a grid-connected PV generator with battery energy storage integration, the study in^10^ offers a thorough method for peak shaving in a commercial building located in Malaysia. The advantages of combining an effective PV- battery energy storage system with the implemented rule-based peak-shaving management algorithm are demonstrated by a comparison of energy and peak demand reduction costs. In order to solve the DRP of a grid-connected residential microgrid system, Chaotic Aquila Optimization has been developed in^11^, where, the primary goal is to optimize the building’s connected appliances’ scheduling patterns in order to reduce total user costs under the dynamic electricity pricing rate. Over time, numerous innovative methods were implemented in the research field of ELD issues. The techniques mentioned in this study were inspired by the natural progress of life^12^. For instance, GA is a widely recognised evolutionary technique that can be employed to tackle the ELD problem. It all starts with the concept of genetics and cell reproduction.

DERs encompass a variety of generators, including those powered by fossil fuels and renewable energy sources^14^. Microgrids are a representation of DERs where the load demand points are spread out over a limited geographical area^15,16^. Microgrids can work in on-grid style or islanded style^16^. But the on-grid style is favoured because of the convenience of buying or selling opportunity from or to the utility. The study in^17^ suggests a novel use of the Pelican Optimization Algorithm for microgrid optimum energy management that takes the DRP into account. Multi-objective optimization is developed in order to optimize the microgrid operators’ benefit and lower the total operating costs, which include the cost of traditional generator fuel and power transaction costs. In order for microgrids to effectively participate in energy trading—which includes energy exchange between microgrids and the utility grid, the authors in^18^ examine both internal and external markets. The energy pricing takes into account two competing goals: the distribution network operator’s desire to maximize its profit from the deployed market and microgrids’ desire to strengthen their economy by lowering their purchase prices and dependency on utility grid. Additionally, this research uses copula-based Monte Carlo methods and hybrid scenarios to evaluate the intermittencies related to correlated renewable energy resources generation, load needs, and PHEV charging demands. In order to address the system uncertainties, the research in^19^ developed an EMS for a multi-microgrid system using the worst-case scenario and a hybrid optimization approach to address the goal. The suggested approach has also been coordinated with an internal market for microgrids’ local energy trading and an IBDR scheme based on pricing elasticity to obtain the best possible economic performance. By establishing dynamic pricing and incentive rates to modify load demands, the DRP strategy successfully reduces microgrid costs. In^20^, a new method for improving a microgrid’s scheduling and energy management is presented. The suggested approach uses an enhanced gradient-based optimization algorithm to reduce energy operation expenses and maximize the production of renewable energy. Comparing scenarios with and without battery storage while accounting for energy exchange with the grid is part of the methodology evaluation.

A hierarchical EMS for several residential energy hubs in the local grid is shown in^21^. The primary goals are to reduce the upstream grid’s peak and maximize financial gain. In this manner, each home energy hub’s energy generation, storage, and purchase/sale are managed by the suggested hierarchical EMS at two levels. With many residential green buildings and a multi-agent system for DER in a neighbourhood grid, an efficient EMS has been demonstrated in^12^. The residential green buildings have a number of electrical and thermal loads with retailers that purchase and sell power to and from residents, as well as controllable and uncontrolled devices by inhabitants and building management systems. The findings demonstrate that the suggested methodology has improved individual residential green building profit and total energy efficiency while managing devices in residential green buildings effectively and promoting DRPs, retailers, and market clearing price reduction. A new method for designing the DRP is presented in^22^, whereby tariffs with time-varying benefits are constructed according to the needs for flexibility. The coordination between the operator of the transmission system and the distribution system is first modelled as a bi-level non-linear programming problem, where the lower-level is day-ahead operational planning of the transmission system and the upper-level is day-ahead operational planning of the distribution system taking into account the schedules received from smart buildings. Because it has decreased the daily costs of the energy and flexibility markets, relieved line congestion, and improved voltage characteristics, the simulation results attest to the fact that the suggested interval-based nested framework has improved the economic, technical, and security aspects of the coordination of transmission and distribution system operators. In order to create an energy-positive/neutral neighbourhood, a power electronic-based house EMS is proposed in^23^ for the neighbourhood network. The structure, features, and EMS of a multi-home energy hub neighbourhood network are categorized. According to the results, under the proposed approach, the home energy hubs provide the multi-home energy hub neighbourhood network with more resources. They so profit more from the sale of excess power. The grid load is also reduced for about 98% of the day. In order to maintain dependability characteristics, EMS based on a multi-agent system controller is designed in^24^ to manage energy, control the voltage, and establish equilibrium between supply and demand in the system. A self-healing hierarchical algorithm is used in the suggested method to regulate agent interaction and ensure the dependability of the smart control system in the event of a fault. The suggested method offers a reliable and stable microgrid control, as shown by theoretical analysis and simulation results for a real-world model. The study in^25^ discusses a two-stage EMS for PHEVs’ contribution to commercial building microgrid DRPs. This work’s primary contribution is the integration of power price uncertainty into a model predictive control-based energy management optimization strategy. In order to increase the resilience of the suggested microgrid while keeping its operating costs as low as possible, the authors in^26^ suggest an ideal battery energy storage management system. The conditional value at risk is integrated into the objective function, and the optimization is accomplished by solving a linear optimization programming problem. The conditional value at risk is used to account for the uncertainty in the intermittent PV system generated power and that in the electricity price. Simulation results reveal that the commercial building microgrid resilience is improved remarkably at a slight increase in the operational cost.

A one-way power flow is conducted for two different scenarios of a microgrid system connected to utility and energy storage systems in^27^, with a 10% increase in the load demand. Cost of producing electrical electricity can be minimised using various methods, such as the Lambda Iteration Technique, Direct Search Method, Lambda logic method, and PSO, as discussed in^28^. Similarly, the consideration of DR on an on-grid microgrid system is factored in, and a network reconfiguration procedure is implemented in^29^. The generating cost of a renewable energy powered microgrid with a redesigned network and IBDR is minimised in^30^ using Hong’s K × m Point Estimation and PSO. In^31^, a PID controller is utilised to minimise the steady state frequency deviation in an autonomous hybrid power system. The DE algorithm is utilised in^32^ to minimise the cost of power production in a cohesive microgrid system that relies on renewable energy sources impacted by DSM policy which involves shifting of elastic loads. The EMS in^33^ establishes the ideal battery size for the energy storage system in addition to reducing emissions and operating expenses. Two crucial factors are also examined in the analysis: the overall daily cost of the battery energy storage system and the distributed generators’ operating and maintenance expenses. In their work, the authors in^34^ present a superior alternative to the same. The paper^35^ demonstrates the application of cosine and sine functions in the field of optimisation. The best calculation of grid-purchased power in a grid-connected photovoltaic/pumped hydro storage system while taking DRP into account is explained in^36^. To determine the values of the choice variables as efficiently as possible, a novel and effective optimization technique called tournament selection-based differential CSA is created. According to simulation studies, determining the power exchange and incentive rate optimally lowers operating costs. Optimal use of DRP lowers operating costs by 5.1 to 14% as compared to the base case (without DRP). CEC2011 is used in^37^ to analyse the resilience of evolutionary approaches. In^38^, the performance of the CSA algorithm is improved by integrating a chaotic approach. An extensive research analysis has been conducted in^39^ regarding the day ahead scheduling in a microgrid system and an energy storage system. An innovative approach using a stochastic expert method^40^ and slime mould algorithm model-based optimisation^41^ is employed to efficiently schedule DERs and ESS, reduced generating costs being the main goal. In^42^, a variety of methods are used to achieve a balanced resolution between minimising the emission of poisonous gases and fuel cost. These methods include the price-penalty factor method, two dynamic economic emission dispatch methods, and the fractional programming method. The CSAJAYA algorithm is utilised to assist in this process. Another research study examines complex and large test systems with a wide range of wind patterns^43^. In order to evaluate the impact of wind power generated by varying wind speeds, a study conducted in^44^ utilised CSAJAYA to model linear, cubic, and quadratic wind profiles for dynamic economic dispatch.

With the active involvement of energy communities and internet data centers^45^, presents a bi-level decentralized approach for structuring the local congestion management market in renewable-based distribution networks. According to simulation results, the suggested mechanism replaces expensive thermal units with contributions from energy communities and internet data centers while protecting the privacy of stakeholders and efficiently providing a sizable amount of the flexible capacities required to reduce network congestion. As a result, local congestion management market expenses are reduced by 61.05%^46^. introduces a novel bi-level approach to controlling the self-healing process in a smart grid that is impacted by energy hubs, hydrogen refueling stations, and EV charging stations. This method increases the speed and dependability of system self-healing by utilizing the combined potential of these prosumers. For the first phase, the smart grid operator communicates necessary nodal capacity to avoid forced load shedding, outlines incentives for smart prosumers, and performs self-healing planning for emergencies. Results demonstrate that the proposed concept reduces forced load shedding by 32.04% and self-healing costs by 17.48% through effective utilization of smart prosumers’ flexible capacities. The tri-layer model for managing a competitive energy market with microgrids and DR aggregators is presented in^47^. In this model, microgrids decide how to participate in the market by taking consumer comfort into account. Through shiftable and curtailable DRPs, the DR aggregator purchases a portion of the customers’ load in the first layer and sells it on the market. The second tier, microgrids, schedule their bids and offers on the market with the goal of maximizing the comfort index and lowering running expenses. Lastly, the market clearing price is found in the third layer. Despite a 12.18% rise in total operating costs, the simulation results demonstrate that the two-objective modelling of the operation problem raises the average comfort index by 5.57%. In order to manage competitive energy and ancillary services markets at the intersection of the distribution and transmission systems^48^, present a two-stage optimization approach. The method considers a wide range of technological, security, and economic aspects. The energy market is included in the first stage of this mechanism’s structure, while ancillary services markets are included in the second. The findings of the simulation show that distribution systems can participate in the spinning reserve market, which lessens the need for expensive transmission systems and lowers system costs by about 10%. Similar works are reviewed in^49^. The system operator uses a strong method to handle operational uncertainty in a bi-level optimization strategy for the decentralized coordination of multi energy communities in competitive gas and electricity markets^50^. While the planning of the natural gas and electricity networks occurs at the lower level, the daily planning of multi energy communities is carried out at the upper level. According to the case study results, the suggested adaptive alternating direction method of multipliers method reduced the solution time by 48.01%^51^. suggests a three-layer risk-averse game theoretic approach for scheduling microgrids and smart buildings and EV fleets. A DRP for smart buildings is created in the initial layer of this technique, and dynamic incentive tariffs are determined by the subscribers’ usage patterns. The scheduling of EV fleets and smart buildings is then carried out in a decentralized environment in the second layer, taking into account their involvement in the intended DRP. Finally, at the third layer, microgrid operators have access to smart building power exchange data so they may schedule their operations accordingly.

### Motivation and contribution addressing the research gap

Researchers have been dynamically occupied in cost-effective approaches for the optimum scheduling of distributed energy sources in a microgrid system, leveraging an assortment of newly established optimization processes. To augment complexity and practicality to their representations, many studies have incorporated innovative restraints, such as the scheduling of PHEV and life cycle calculations of battery ESS. Furthermore, some studies have implemented load-shifting policies, which reorganize elastic or flexible loads to periods when utility rates are lesser. This tactic aids in reducing peak loads, improving the load factor, and enhancing the total efficiency of the microgrid system. Still, much of these cutting-edge investigations have neither suggestively focused on plummeting the pollutants discharged by DERs, nor have it prioritized pollutant reduction as a primary aim. Addressing this research gap, the present study introduces an IBDR mechanism, rewarding microgrid customers for reducing their energy consumption during peak hours. This load reduction achieves two key objectives: first, it lowers the generation costs of the microgrid system, and second, it reduces the power production required from DERs, thereby decreasing emissions. Additionally, the study suggests a trade-off solution that balances cost minimization and emission reduction, giving equal importance to both objectives. To sum up, the key contributions of the paper are as follows:

- IBDR is availed for restructuring the load demand for different levels of customer participation.
- Exhaustive techno-economic scenario analysis is performed with and without IBDR to evaluate the impact of various grid participation and market pricing approaches.
- Cost comparative analysis of the results obtained with other demand side management policies available in literature.
- Weighted economic emission dispatch is evaluated in presence and absence of IBDR to obtain a balanced compromised resolution between emission and cost of generation.

### Organization of the paper

The subsequent sections of the manuscript are organised as follows. A description of the fitness function to be reduced is given in section “Formulation of fitness function”. It also discusses how to strike a compromise in the DR based Microgrid Energy Management for the systems under analysis between equality and inequality constraints. Section “Methodology” explains a low voltage grid connected microgrid system that is considered for the simulation. The presented work is finally summarised in section “Conclusive remarks and future course of action”.

## Formulation of fitness function

Equation (1) represents mathematically the cost of producing electrical power by DERs and the energy market rate that power utilities pay^52,53^, in which *genr* stands for the number of generators, *tm* for time, *CCG* for the generator’s cost coefficients, *PG* for the generators’ power output, *IG* for the generator’s status *(i.e.*,* 1 for ON and 0 for OFF)*,* SUC* for the start-up cost, *SDC* for the shutdown cost, *mp*_*grid*_ for the grid’s electricity market price, and *PC*_*Grid*_ for the grid’s electrical power consumption and supply. In situations where the grid utilises a specific hourly rate to buy and sell electricity from the microgrid, the rate is calculated as the overall cost of the microgrid$~{C_{ng}}$, That depends on how much power the utility buys and how much power it sells, as indicated by (2)^52^. The formula for the rate of importing electrical power, ${C_{buy}}$, and the rate of importing electrical power, ${C_{sell}}$, is given by Eq. (3)^52^ and (4)^52^. The utility sets the tax, which changes according to the percentage value of the grid-set energy market price.

1 $${\text{ECD}}=Min\mathop \sum \limits_{{tm}} \mathop \sum \limits_{{genr}} \left[ {CC{G_{genr}}\left( {PG_{{genr}}^{{tm}}} \right)IG_{{genr}}^{{tm}}+SUC_{{genr}}^{{tm}}+SDC_{{genr}}^{{tm}}} \right]+\mathop \sum \limits_{{tm}} {C_{ng}}_{{}}^{{tm}}PC_{{grid}}^{{tm}}$$

2 $${C_{ng}}={C_{buy}}+{C_{sell}}$$

The grid is weighing three different approaches to carry out power purchasing and selling to and from the microgrid system. The first technique takes into account the electricity market price *(mp*_*grid*_*)* as the same for purchasing and selling power. The second approach considers various rates for buying and selling power, as shown in Eq. (3). Equation (4) in the final method calculates the taxable proportion of the cost price by considering the selling price.

3 $${C_{buy}}=\mathop \sum \limits_{{tm}} mp_{{grid}}^{{tm}}PC_{{grid}}^{{tm}}{\text{~}}if{\text{~}}PC_{{grid}}^{{tm}}>0$$

4 $${C_{sell}}=\mathop \sum \limits_{{tm}} \left( {1 - tax} \right)mp_{{grid}}^{{tm}}PC_{{grid}}^{{tm}}{\text{~}}if{\text{~}}PC_{{grid}}^{{tm}}<0$$

To account for the- atmospheric emission (specifically carbon dioxide) caused from the fossil fuel-based generation, *CFI*, is calculated as per (5)^54^, where ${E_j}$ is the carbon dioxide factor for generator *j*, ${E_{grid}}$ is the carbon dioxide factor for grid, *PC*_*grid*_ is the grid generated power.

5 $$CFI=\mathop \sum \limits_{{tm}}^{{24}} \mathop \sum \limits_{{j=1}}^{n} \left( {{E_j}PG_{{genr,j}}^{{tm}}+{E_{grid}}PC_{{grid}}^{{tm}}} \right)$$

To account both for the objective functions given by (1) and (5), a metric was developed in^54^ as per (6), called WEED. $\mu$ lies in between 0 and 1, $EC{D_{min}}$ and $CF{I_{min}}$ are the optimum values of (1) and (5) respectively. $EC{D_{max}}$ and $CF{I_{max}}$ are collected by substituting the optimal parameters of $EC{D_{min}}$ in (5) and $CF{I_{min}}$ in (1), respectively.

6 $$WEED=\mu \left[ {\frac{{ECD - EC{D_{min}}}}{{EC{D_{max}} - EC{D_{min}}}}} \right]+\left( {1 - \mu } \right)\left[ {\frac{{CFI - CF{I_{min}}}}{{CF{I_{max}} - CF{I_{min}}}}} \right]$$

Equation (7) is an objective function developed taking the load and generating unit limitations into account^52,53^. The constraints for the grid are given as per (8)^52,53^. The constraints for the generators are given as per (9)–(11)^52,53^, where *T*^*on*^ and *T*^*off*^ are the on time and off time of the generators. *I* is the binary index where 1 means on and 0 means off.

7 $$\mathop \sum \limits_{{tm}} \mathop \sum \limits_{{genr}} PG_{{genr}}^{{tm}}+PC_{{grid}}^{{tm}}=\mathop \sum \limits_{d} \mathop \sum \limits_{{tm}} L_{d}^{{tm}}$$

8 $$- PG_{{genr}}^{{min}} \leqslant PC_{{grid}}^{{tm}} \leqslant PG_{{genr}}^{{max}}$$

9 $$PG_{{genr}}^{{min}} \leqslant PG_{{grid}}^{{tm}} \leqslant PG_{{genr}}^{{max}}$$

10 $$T_{{gen}}^{{on,~tm}} \geqslant ON{T_{grid}}\left( {I_{{grid}}^{{tm}} - I_{{grid}}^{{tm - 1}}} \right)$$

11 $$T_{{gen}}^{{off,~tm}} \geqslant OFF{T_{grid}}\left( {I_{{grid}}^{{tm}} - I_{{grid}}^{{tm - 1}}} \right)$$

The relationship between hourly wind power and hourly wind speed can be mathematically modelled linearly as per (12)^44^, the wind speed is represented by *s*, $s_{j}^{i}$, $s_{j}^{r}$ and $s_{j}^{o}$ wind speed at cut in, rated, and cut out of the *j*th wind generator unit respectively and $wp_{j}^{r}$ is the rated wind power.

12 $$wp_{j}^{{tm}}=\left\{ {\begin{array}{*{20}{c}} {wp_{j}^{r}\left( {\frac{{s - s_{j}^{i}}}{{s_{j}^{r} - s_{j}^{i}}}} \right)~~~~~~if~s_{j}^{i} \leqslant s \leqslant s_{j}^{r}} \\ {wp_{j}^{r}~~~~~~~~~~~~~~~~~~~~~~~~~if~s_{j}^{r} \leqslant s \leqslant s_{j}^{o}} \\ {0~~~~~~~~~~~~~~~~~~~~~~~~~~~if~0 \leqslant s \leqslant s_{j}^{i}} \end{array}} \right.$$

In order to account for the unpredictable and random characteristics of wind power, it is necessary to include uncertainty into the projected values. The uncertainty can be mathematically modelled as per (13)^44^, where$~d\left( {wp} \right)$ is the standard deviation of the wind power as per (14)^44^, $wp_{{un}}^{{tm}}$ is the wind power including uncertainty, $wp_{{fc}}^{{tm}}$ is the estimated wind power and ${\xi _1}$ is random normal distribution function having a mean value of 1 and 0 standard deviation.

13 $$wp_{{un}}^{{tm}}=d\left( {wp} \right){\xi _1}+wp_{{fc}}^{{tm}}$$

14 $$d\left( {wp} \right)=0.8\surd wp_{{fc}}^{{tm}}$$

The percentage of utilization is represented by (15)^45,46^.

15 $$PU=\frac{{\mathop \sum \nolimits_{{tm=1}}^{{24}} wp_{{gen,~grid}}^{{tm}}}}{{24*wp_{{gen,~grid}}^{{max}}}}$$

### Fiscal model of the load for the DRP

**Fig. 1:**
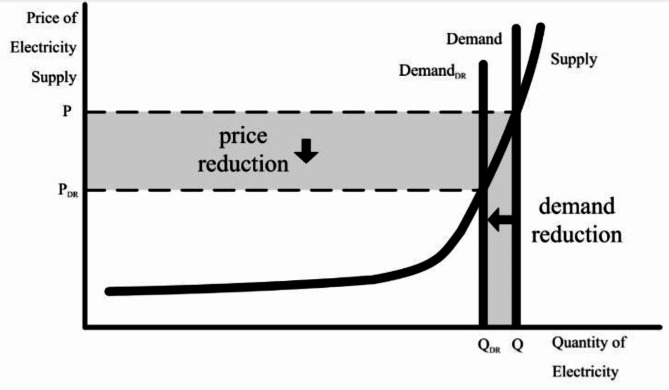
Effect of DR in load demand and price reduction^48^.

By employing the theories of demand price electricity, the fiscal model of the load due to the DRP can be obtained. The price elasticity matrix plays a crucial role in curtailing the loads thereby reducing the electricity price additionally providing incentives as depicted in Fig. 1. The particulars of the model are explained in^29,55,56^. Thus, its derivation is not dealt with here. The load, taking part in the DRP is modified as per (16)^55^, where $$L_{{cj}}^{{DRp}}\left( {tm} \right)$$ is the *j*th bus load due to DRP for case *c* in time *tm*, $$\xi$$ is the amount of load (in percent) taking part in DRP, $$L_{{cj}}^{{ini}}\left( {tm} \right)$$ is the initial value of load demand for *j*th bus for *c* case in *tm* time, and $${\Upsilon _0}\left( {tm} \right){\text{~and~}}$$$$\Upsilon \left( {tm} \right)$$ are the initial and spot electricity rate for hour *tm* respectively. The consumers have the option to regulate their loads as per $$\Upsilon \left( {tm} \right)$$. This means that the consumers can transfer their load from a time period having higher $$\Upsilon \left( {tm} \right)$$ to a period having lower $$\Upsilon \left( {tm} \right)$$. For this, the customer is provided with an incentive $$IN\left( {tm} \right)$$ for participation in the incentive based DRP and hence agrees to shift or cut the load for *tm* duration (in hours). The cross and self-elasticities are denoted respectively by $$E\left( {tm,m} \right){\text{~and~}}E\left( {tm,tm} \right)$$. The values of cross and self-elasticities are taken to be positive and negative correspondingly.

16 $$L_{{cj}}^{{DRp}}\left( {tm} \right)=\left[ {\begin{array}{*{20}{c}} {E\left( {tm,tm} \right)\left( {\frac{{\Upsilon \left( {tm} \right) - {\Upsilon _0}\left( {tm} \right)+IN\left( {tm} \right)}}{{{\Upsilon _0}\left( {tm} \right)}}} \right)+} \\ {\mathop \sum \limits_{{m=1;m \ne tm}}^{{24}} E\left( {tm,m} \right)\left( {\frac{{\Upsilon \left( m \right) - {\Upsilon _0}\left( m \right)+IN\left( m \right)}}{{{\Upsilon _0}\left( m \right)}}} \right)+1} \end{array}} \right]\xi L_{{cj}}^{{ini}}\left( {tm} \right)$$

For the scenarios in a price-based DRP paradigm, $${\Upsilon _0}\left( {tm} \right){\text{~and~}}$$$$\Upsilon \left( {tm} \right)$$ have different values. If the value of the difference between the spot and initial electricity rate is positive, i.e. $$\Upsilon \left( {tm} \right) - {\Upsilon _0}\left( {tm} \right)>0$$, it is equivalent to the incentive based DRP scenario. The loads are displaced from a higher spot price slot to a lower one. In the present work, the values of $${\Upsilon _0}\left( {tm} \right){\text{~and~}}$$$$\Upsilon \left( {tm} \right)$$ are taken as same to simulate a DRP with incentive feature. In this DRP, the loads are curtailed as per (17)^55^, where $$\Delta L_{{cj}}^{{DRp}}\left( {tm} \right)$$ is the amount of curtailed load at bus *j* for case *c* at time *tm*. The aggregated load at bus *j*, $${L_j}$$, in case *c* at time *tm* is denoted by (18)^29^.

17 $$\Delta L_{{cj}}^{{DRp}}\left( {tm} \right)=~\xi L_{{cj}}^{{ini}}\left( {tm} \right) - L_{{cj}}^{{DRp}}\left( {tm} \right)$$

18 $${L_j}=\left( {1 - \xi } \right)L_{j}^{{ini}}\left( {tm} \right)+L_{j}^{{DRp}}\left( {tm} \right)$$

## Methodology

### Test system considered

**Fig. 2:**
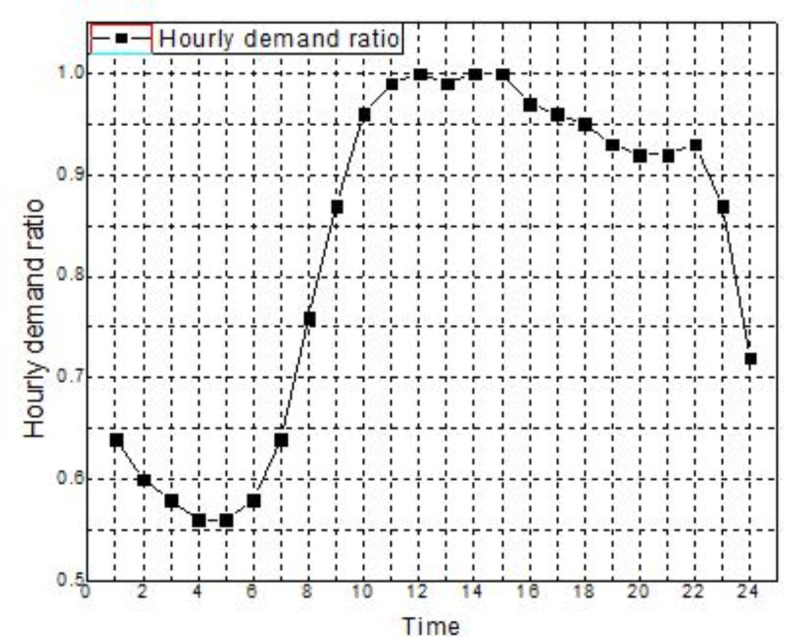
Ratio of load demand in accordance with peak demand^57^.

**Fig. 3:**
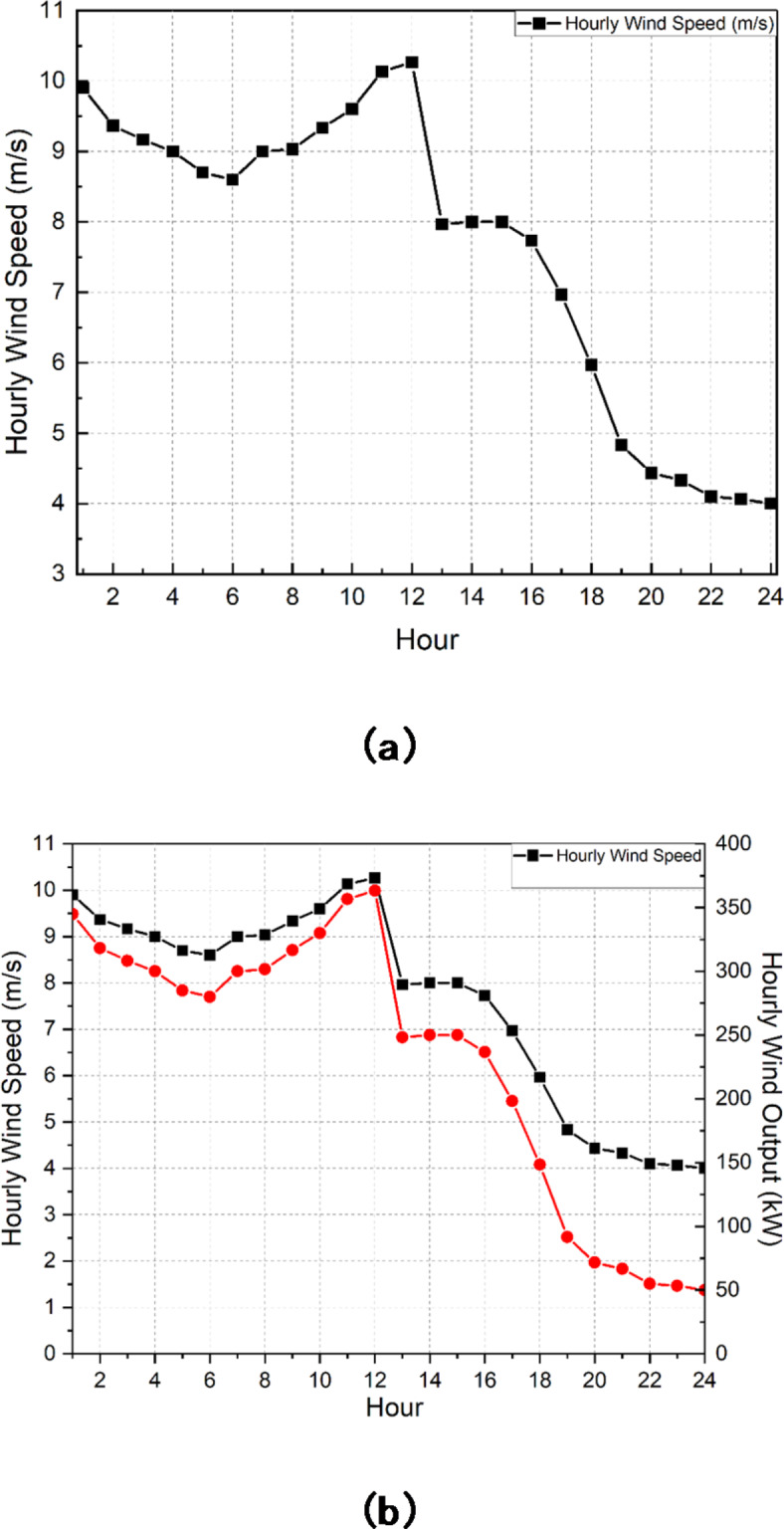
**(a)** Forecasted wind speed on hourly basis^57^. **(b)** Forecasted wind power on an hourly basis in correspondence with the speed.

**Fig. 4:**
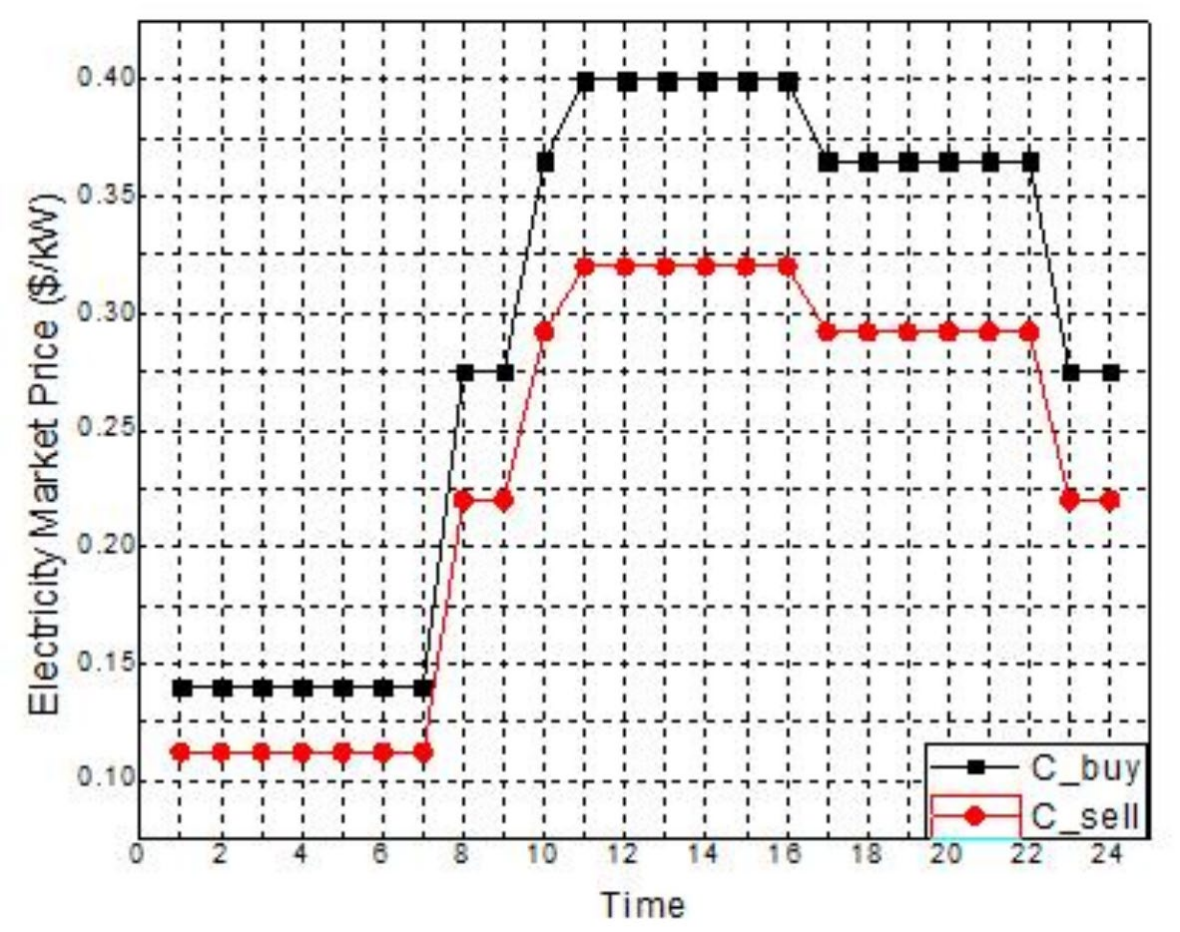
Hourly electricity market price^57^.

The primary aim of the paper is to achieve a well-adjusted negotiated resolution between minimal values of pollutants emission and cost of generation. The work is segregated into three levels. The primary level emphasizes the beneficial impact of implementing IBDR on the dynamic distribution system, namely the microgrid system in this instance. A comprehensive and thorough techno-economic study under different grid pricing and involvement approaches are considered for six different scenarios in the second level and a comparison is being made among the scenarios. Finally, on the third level, an assessment is being conducted between two distinct ways of combining economic emission dispatch in order to determine the methodology that yields the lowest negotiated value for both emission and generation cost. The test system taken for this paper is an on-grid microgrid system energized by MT, biomass, NGFC and CHP system as given in^57^. The parameters relating to these DERs are tabulated as per Table 1. The microgrid system has a peak demand of 3715 kW. Figure 2 illustrates the ratio of load demand relative to peak demand. There is a capacity of 500 kW that is rated for the WT. The wind speed values used for the rated, cut in, and cutoff points are 13 m/s, 3 m/s, and 25 m/s, correspondingly. The wind speed that is projected to occur every hour is illustrated in Fig. 3a. The predicted wind power on an hourly basis, based on the wind speed, is shown in Fig. 3b using mathematical Eq. (13). Figure 4 illustrates the dynamic TOU based energy market price bid used by the grid to conduct power transactions with the microgrid system. Table 2 presents a comprehensive analysis of seven scenarios that are investigated to evaluate the whole techno-economic performance of the microgrid system. Table 3 presents the price elasticity values for different degrees of load demand. The research uses the DE technique, as described in^58^, for optimisation purposes. It is coded and implemented in MATLAB R2021b software on a Core i7 2nd Gen processor, 16GB RAM laptop. The Crossover Ratio is tuned at 0.25 and the Mutation Factor is tuned at 0.75. The maximum number of iterations and population size considered for the study is 1000 and 80 respectively.

**Table 1:** DER parameters^57^.

DG units	MT	CHP	NGFC	BIOMASS	GRID
Efficiency	0.3	0.35	0.35	0.29	Not applicable
P_max_ (kW)	600	800	800	1000	800
Emission (kg/kWh)	0.724	0.408	0.336	0.003	0.547
Cost of O&M ($/kWh)	0.00587	0.00419	0.00419	0.01258	Not applicable
P_min_ (kW)	100	100	100	100	− 800
Cost of the fuel ($/kWh)	0.021	0.027	0.027	0.063	Figure 4

**Table 2:** Details of different scenarios considered.

Scenario	Brief terminology	Complete explanation
1	Ideal Scenario	In order to distribute the load demand, every DER and grid will actively participate. Grid pricing is same for both buyers and sellers
2	Absence of RES	Ignoring the wind system’s contribution, it’s identical to Scenario 2
3	Passive Grid	The grid’s only function is to provide (sell) electricity as a backup^52^
4	Taxable price is 20%	Equations (3) and (4) governs the Cost function of grid; Percentage of tax is considered as 20%^52,57^
5	Taxable price is 50%	Equations (3) and (4) governs the Cost function of grid; Percentage of tax is considered as 50%^52,57^
6	Dissimilar CP/SP	Figure 4 governs the electricity price^52^

**Table 3:** Price elasticity values^29^.

	Off-Peak	Valley	Peak
Peak	0.016	0.012	− 0.1
Valley	0.01	− 0.1	0.012
Off-Peak	− 0.1	0.01	0.016

### Effects of IBDR implementation

This is the first stage wherein the load demand model was reorganized for different levels following the incentive-based IBDR strategy mentioned above. Considering 30 to 40% of the microgrid customers were willing to participate in the DR model, the optimal incentive value was carefully evaluated. The maximum incentive cost was limited to $250 at the end of the day and it was also maintained that not more than 5% of the entire load demand was to be curtailed by the customers. Abiding by these constraints the incentive value was found to be 6 cents/kW. The advantages of implementation as displayed in Table 4 are as follows:

- For 30% of customers participating in IBDR program, the incentive cost at the end of the day was $185. A 6% decrease in peak demand from 3715 to 3486 kW was observed. Moreover, the load factor increased from 0.8292 to 0.8521. Only 3.5% of the total load was curtailed by the customers.
- Assuming a 40% participation of the microgrid customers in the IBDR program, the incentive cost at the end of the day was $247. An 8% decrease in peak demand from 3715 to 3410 kW was observed. Moreover, the load factor increased from 0.8292 to 0.8604. Only 4.7% of the total load was curtailed by the customers.

**Fig. 5:**
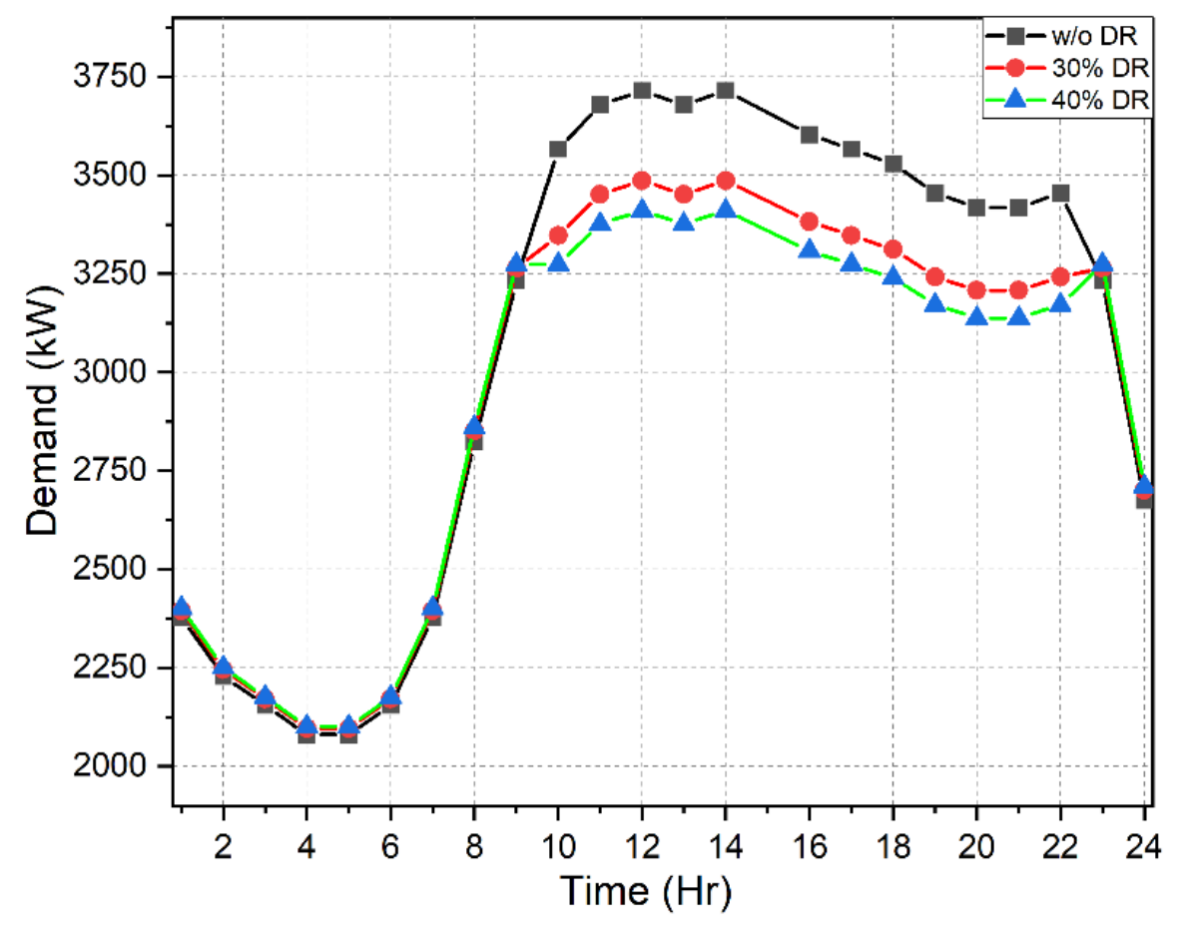
Load profile structure before and after IBDR implementation.

**Fig. 6:**
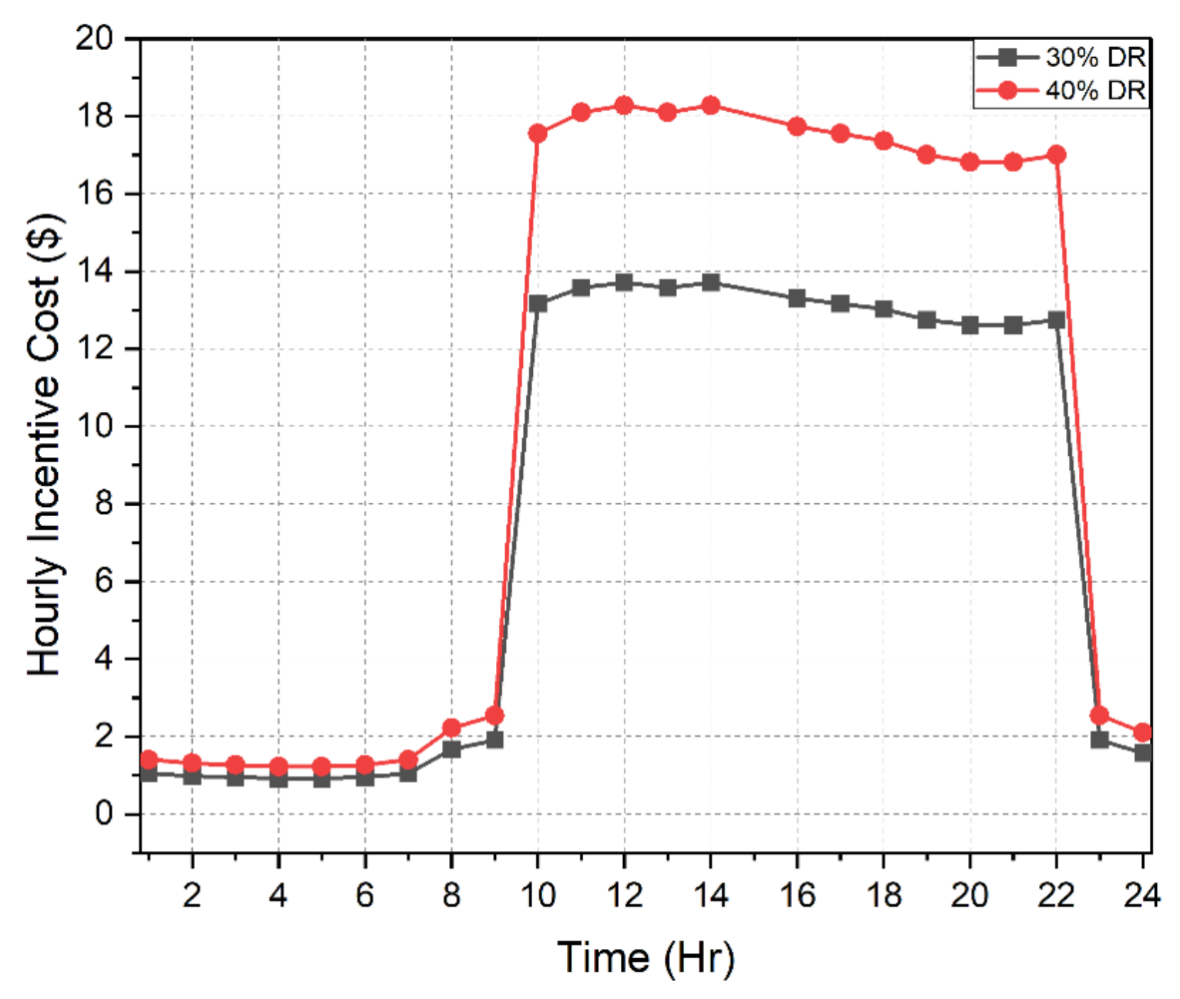
Hourly incentive costs for 30% and 40% IBDR participation.

The forecasted load demand model along with the reorganized load demand in view of 30% and 40% DR participation level is depicted in Fig. 5. The decline in the peak demand can be easily noticed in the figure. Figure 6 shows the hourly incentive costs which add up to $185 and $ 247 for 30% and 40% DR participation level respectively.

**Table 4:** Load demand analysis with and without IBDR implementation.

Stage 1	Effects of IBDR implementation				
	Peak demand (kW)	Incentive cost ($)	Peak reduction (%)	Load factor	Load curtailed (%)
w/o DR	3715	0	0	0.8292	0
30% DR	3486.5	185.5963	6.1521	0.8521	3.5569
40% DR	3410.3	247.4617	8.2027	0.8604	4.7426

### Exhaustive techno-economic analysis

**Table:** 

Scenario 1	Ideal Scenario: In this case, the hourly load needs are being shared actively by all the DERs. Under this scenario, the grid maintains a constant rate of power sales to the microgrid (C_sell in Fig. 4). The cost of generation for this scenario is found to be $8326. This exemplifies the benefits of a microgrid system in which distributed DERs meet load demand rather than depending only on the grid. Even with the restructured load demand and 30% DR participation level, the cost of generating reduced to $7468. This included an incentive cost of $185 to be paid to the customers. In addition, taking into account the restructured load demand with a 40% DR participation level reduced the generating cost to $7182. This included an incentive cost of $247 to be remunerated to the customers. Figures 7, 8 and 9 depict the load sharing model of the DERs in three scenarios: without DR, with 30% DR, and with 40% DR, where the objective is to minimize the cost of production. The image displays the active transactions of electricity being bought and sold on the grid. This scenario is taken as a reference for comparison with other scenarios
Scenario 2	*Absence of RES*: The involvement of the wind turbine is not taken into account for this scenario. The wind turbine’s total yield, shown in Fig. 3b, accounts for 5525 kW, which is approximately 7% of the microgrid system’s total load demand. The cost of generation for this scenario is obtained as $9857 that is nearly 18% increase than the ideal scenario. Restructured load demand model owing to the DR participation have diminished the generation cost in this scenario too
Scenario 3	*Passive Grid*: During this scenario, the grid’s involvement is focused on meeting the demand for electricity. Specifically, the grid will only provide power when the DERs are unable to provide the required amount of electricity. The grid will behave as a standby mode for the rest of the time. Mathematically, this scenario may be represented by establishing the minimum value of the grid as zero. Since the grid is not actively involved in the process of selling and buying electricity, it is logical that the cost of power production in this situation ($8571) would be higher than in the ideal scenario ($8326). Figure 10 illustrates the hourly output of DERs in Scenario 3 with a 40% DR when the generating cost is minimised. It appears that the grid is supplying electricity from 10 to 22 h. No negative value of grid is observed in the figure unlike Figs. 7, 8 and 9
Scenario 4 & Scenario 5	*Taxable SP*: The grid sells and buys power at different costs as mathematically shown in Eqs. (3) and (4). The tax for Scenario 4 and Scenario 5 is 80% and 50% respectively. The cost of generation obtained with 80% tax is $8475 and with 50% tax is $8562. In both situations, the generation costs exceed the ideal scenario, regardless of the presence or absence of DSM.
Scenario 6	*Dissimilar SP/CP*: Fig. 4 depicts the electricity market price variation. The cost of generation in this scenario is more than the ideal one

**Table:** 

Overall observation	The lowest generation cost is obtained for the ideal scenario cost in comparison to other scenarios. This is true for both with and without the involvement of IBDR policy
	The cost of electricity production decreased in all scenarios as the amount of customers participating in DR involvement increased. This demonstrates the usefulness of load restructuring via the use of DR as a cost-effective way for operating microgrids
	The generation cost which involves incentives to be paid to the customers, is still less than the generation cost which considers load shifting policy-based DSM without rewarding the customers as mentioned in literature^32^
	visual depiction of Table 5 may be seen in Fig. 11, which also contains the information that was previously discussed

**Table 5:** Generation costs ($) for diverse scenarios and DR participation levels.

DR level	Stage 2	Ideal scenario	Without RES	Passive grid	20% taxable SP	50% taxable SP	Different SP/CP
0%	**Gen Cost**	**8326**	**9857**	**8571**	**8475**	**8562**	**8475**
40% DSM^32^	**Gen Cost**	7311	8781	7744	7513	7690	7513
30%	Gen Cost	7282.9888	8813.5882	7625.9383	7475.51	7611.161	7475.51
	In Cost	185.59	185.59	185.59	185.59	185.59	185.59
	Total	**7468.5788**	**8999.1782**	**7811.5283**	**7661.1**	**7796.751**	**7661.1**
40%	Gen Cost	6935.1	8465.7002	7425.0792	7191.9718	7391.957	7191.9718
	In Cost	247.4617	247.4617	247.4617	247.4617	247.4617	247.4617
	Total	**7182.5617**	**8713.1619**	**7672.5409**	**7439.4335**	**7639.419**	**7439.4335**

Significant values are in [bold].

**Fig. 7:**
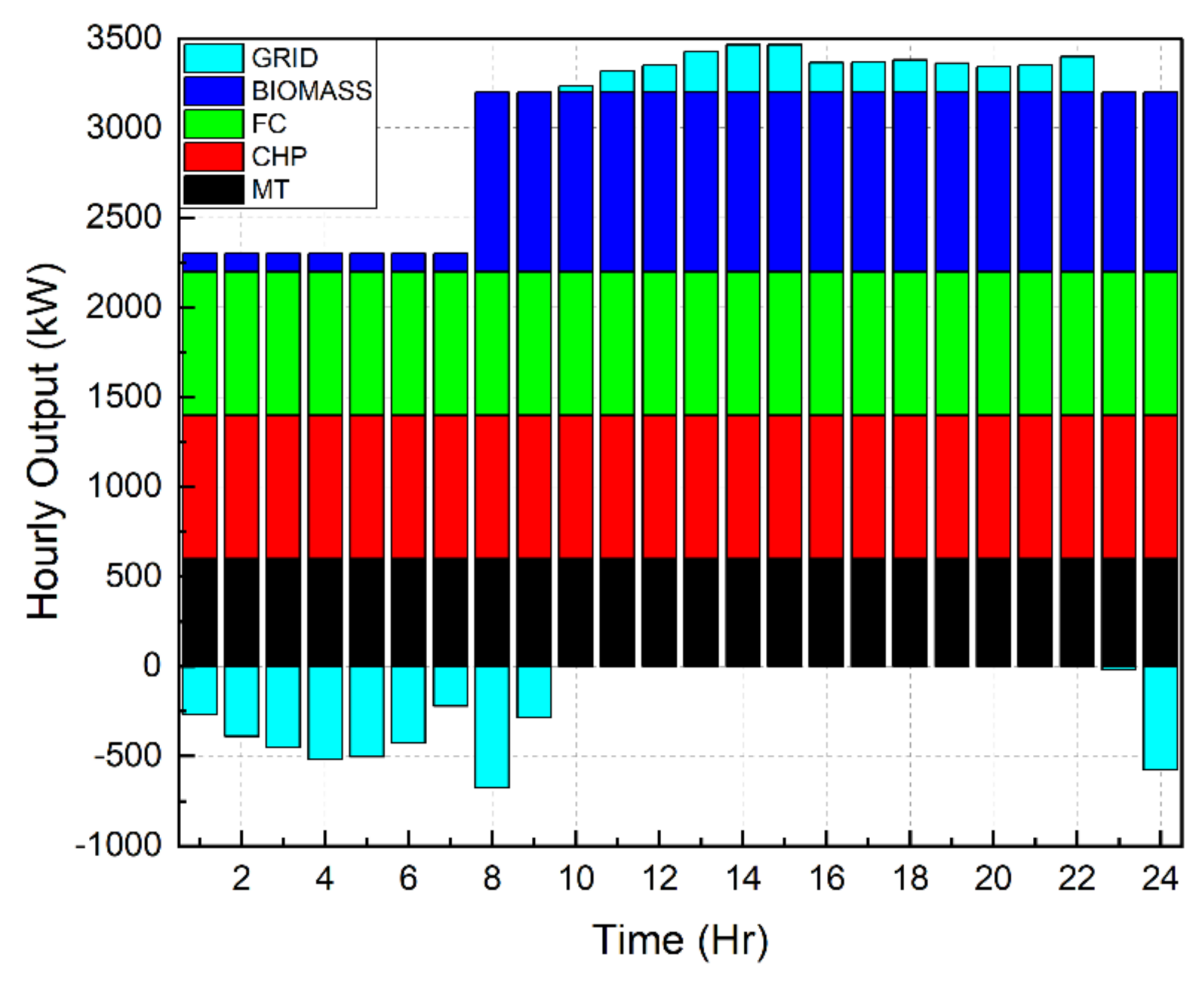
Hourly outputs for DERs during scenario 1.

**Fig. 8:**
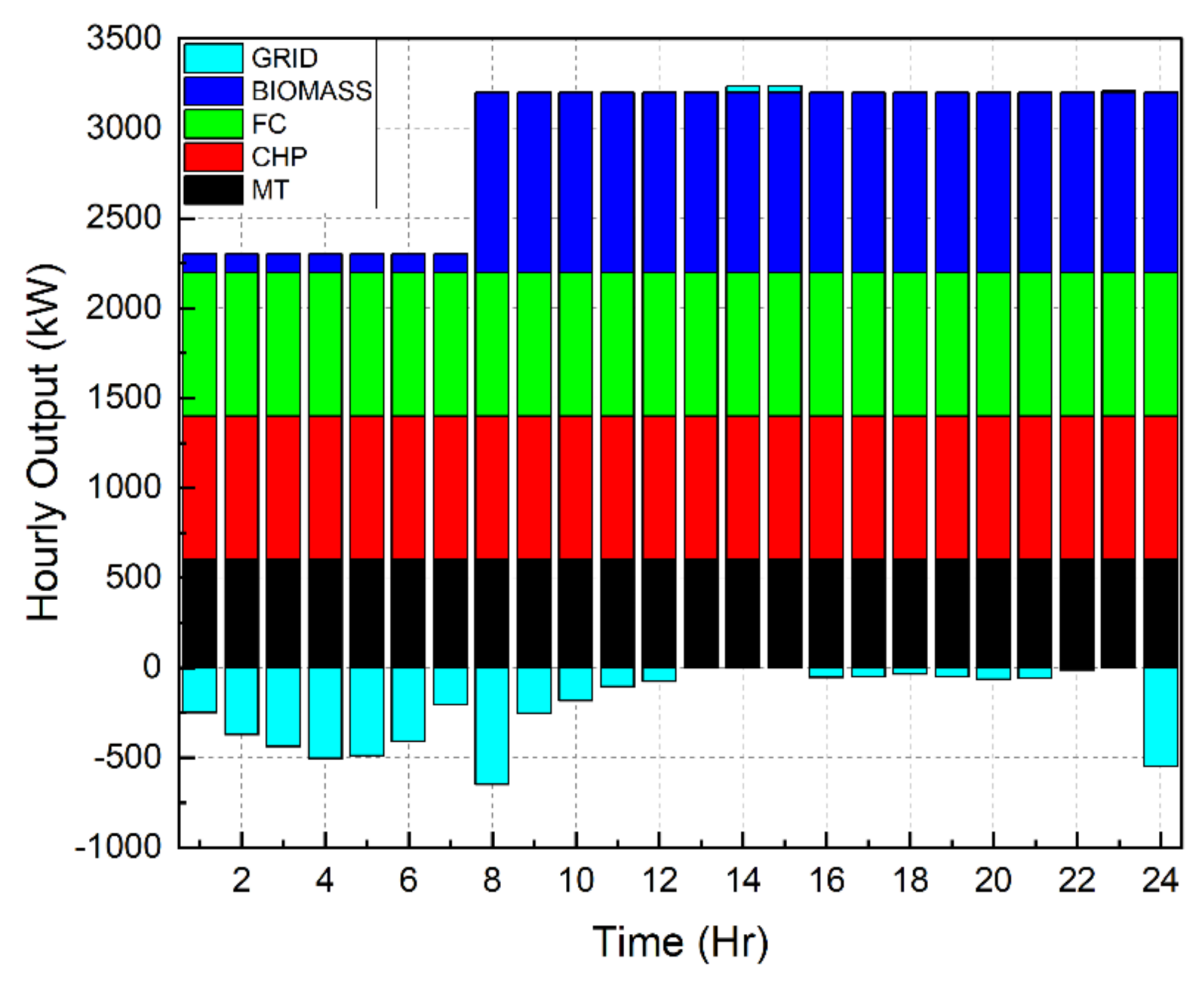
Outputs for DERs on hourly basis during scenario 1 with 30% DR.

**Fig. 9:**
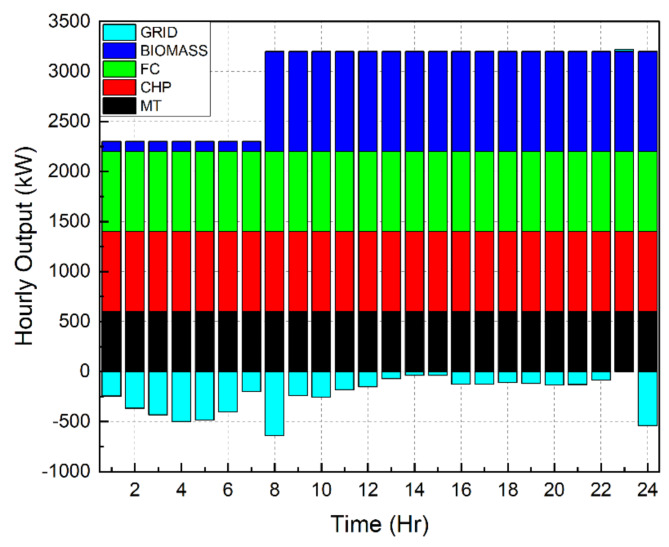
Hourly outputs for DERs during Scenario 1 with 40% DR.

**Fig. 10:**
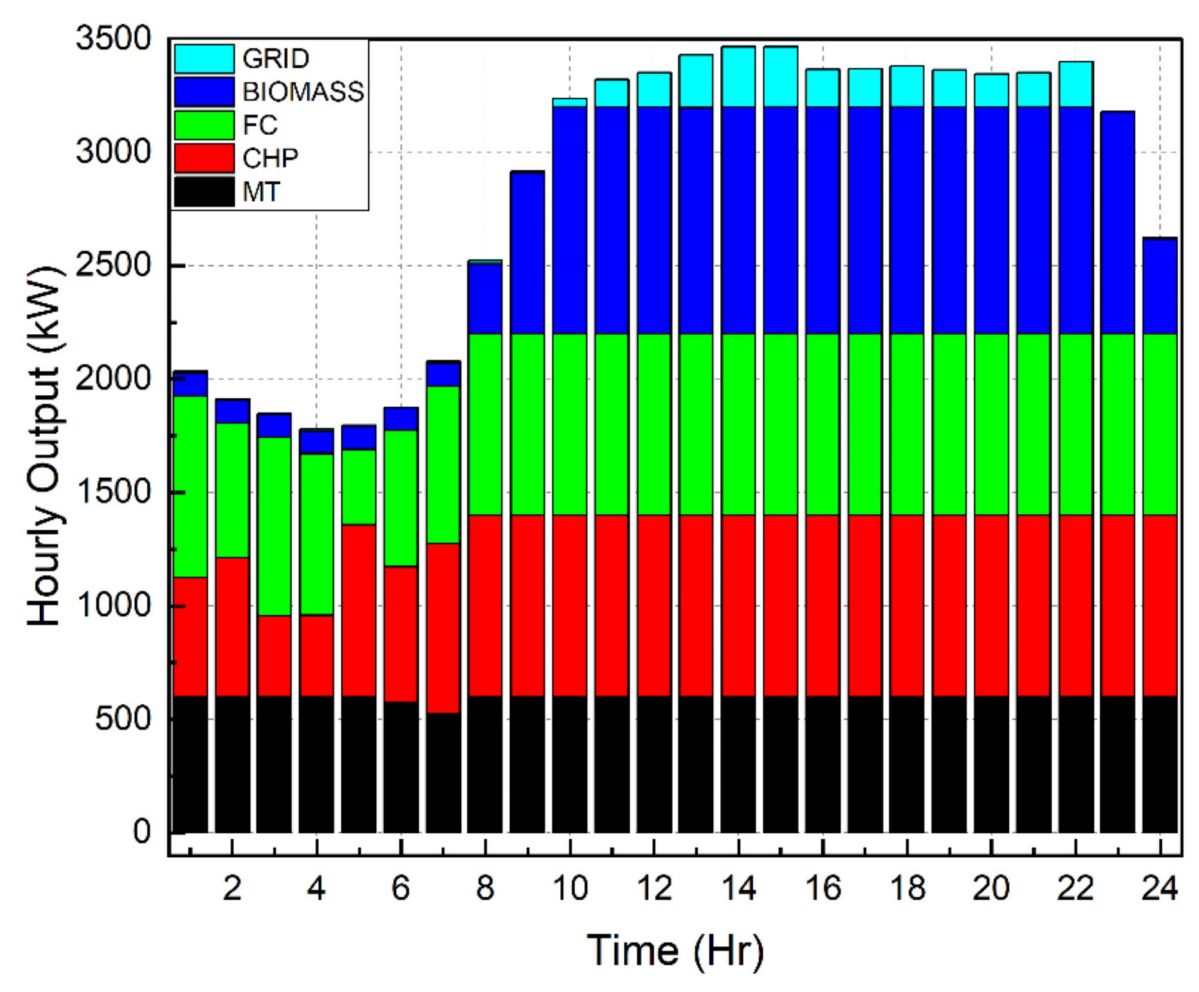
Hourly outputs for DERs during scenario 3.

**Fig. 11:**
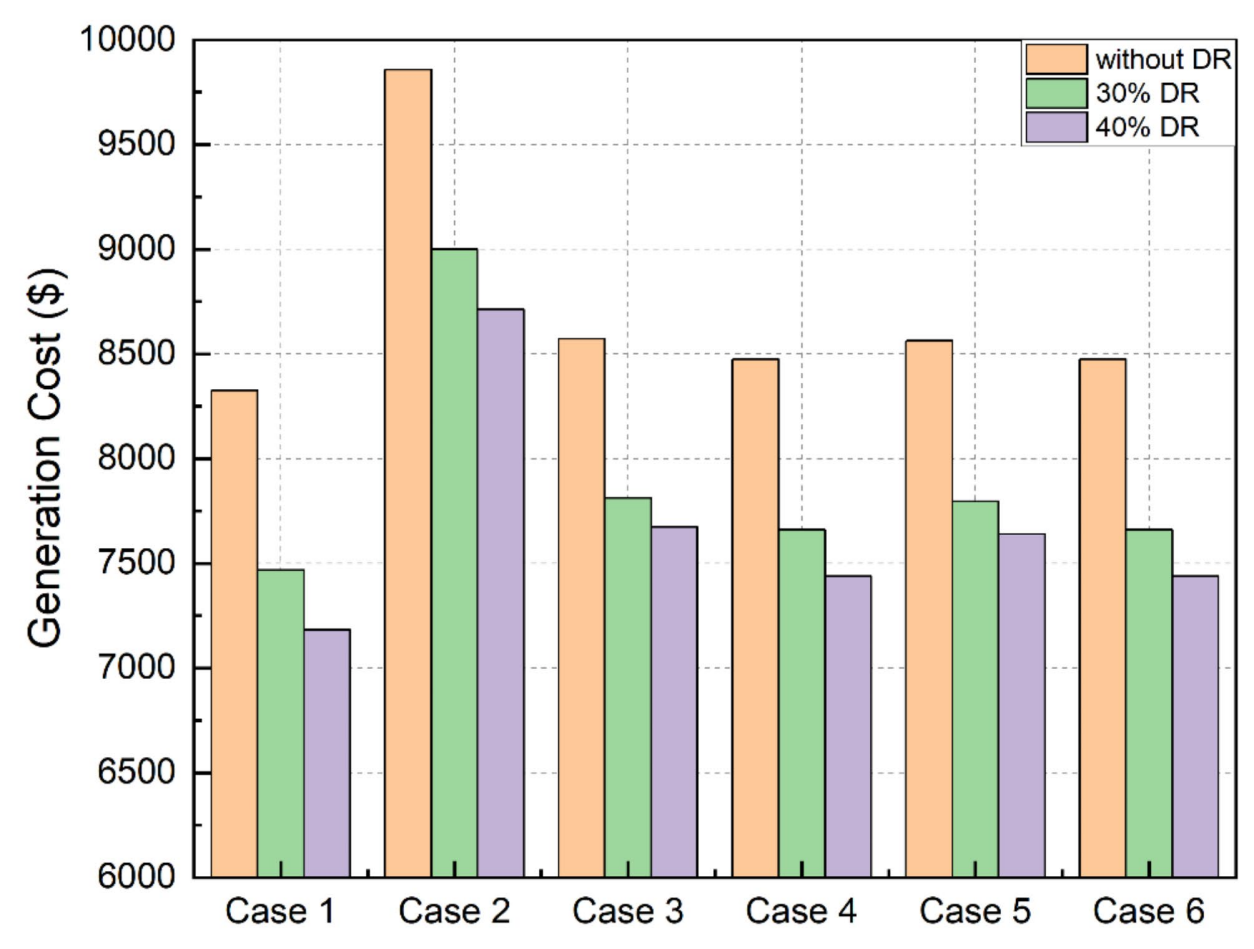
Cost comparative analysis.

### Balanced solution between emission and generation cost

**Fig. 12:**
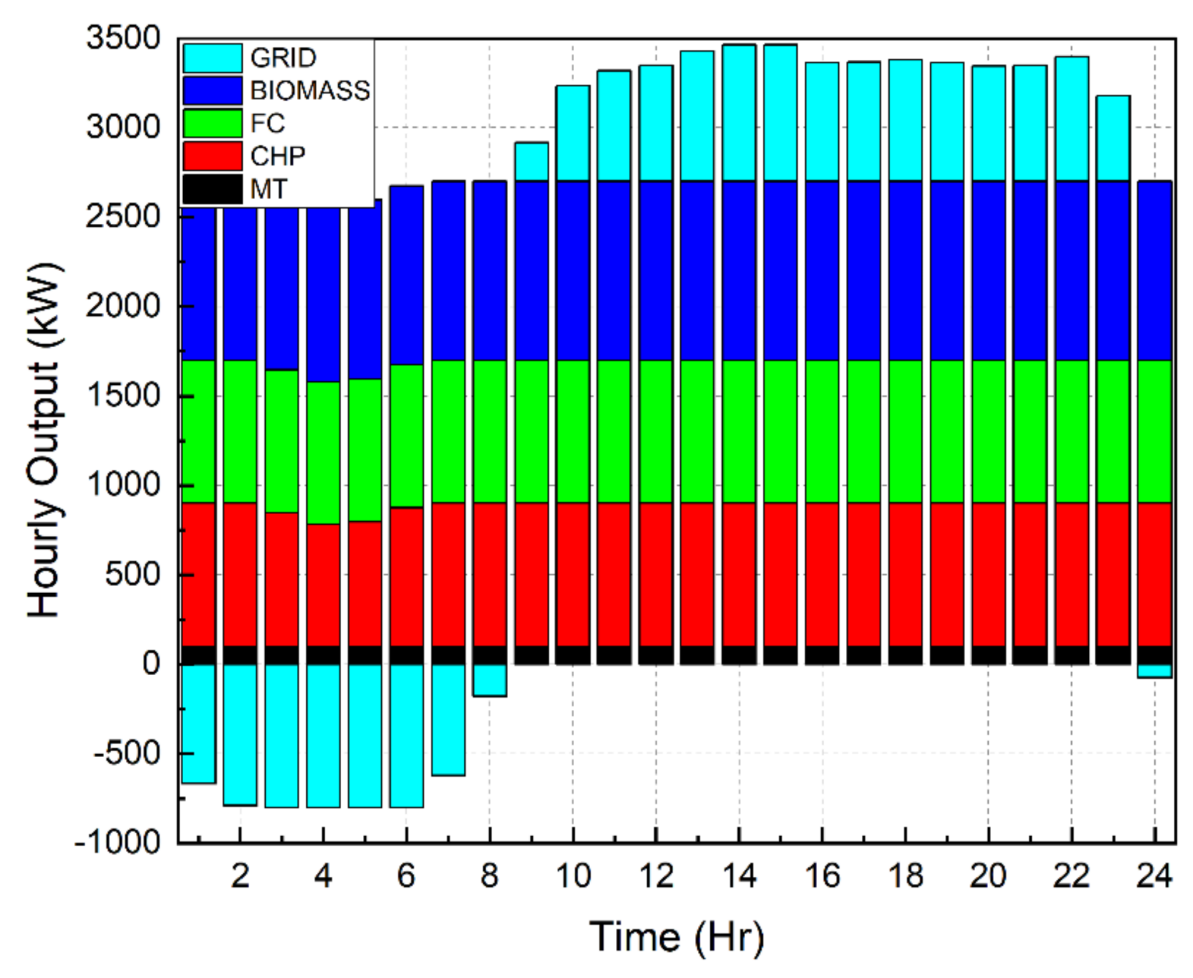
Sharing of loads among DERs for CFI minimization (without DR).

In the final stage of the paper, a balanced resolution between emission and generation cost is evaluated by minimizing the WEED equation as mentioned in section “Formulation of fitness function”. To start with, the emission equation represented by Eq. (5) is minimized to reduce the CFI for different load demand models. It was observed that the emission was 18,107 kg, 16,660 kg and 16,178 kg for load demands without DR, with 30% DR and with 40% DR respectively. This decrement in emission for different load demand models happened due to the curtailment of loads. Figure 12 shows the distribution of load amongst DERs when CFI was minimized without DR participation. It can be seen that the load sharing is totally different from Fig. 7 which emphasized in minimizing the cost of generation for the system.

**Table 6:** WEED evaluation table for µ = 0.5.

Stage 3	Without DR	30% DR	40% DR
WEED value	0.2633	0.2626	0.2623
In Cost ($)	0	185.59	247.4617
Gen Cost ($)	9134.8	8087.4	7738.3
Total Cost ($)	9134.8	8272.99	**7985.7617**
Emission (kg)	19,612	18,165	**17,683**

Significant values are in [bold].

**Fig. 13:**
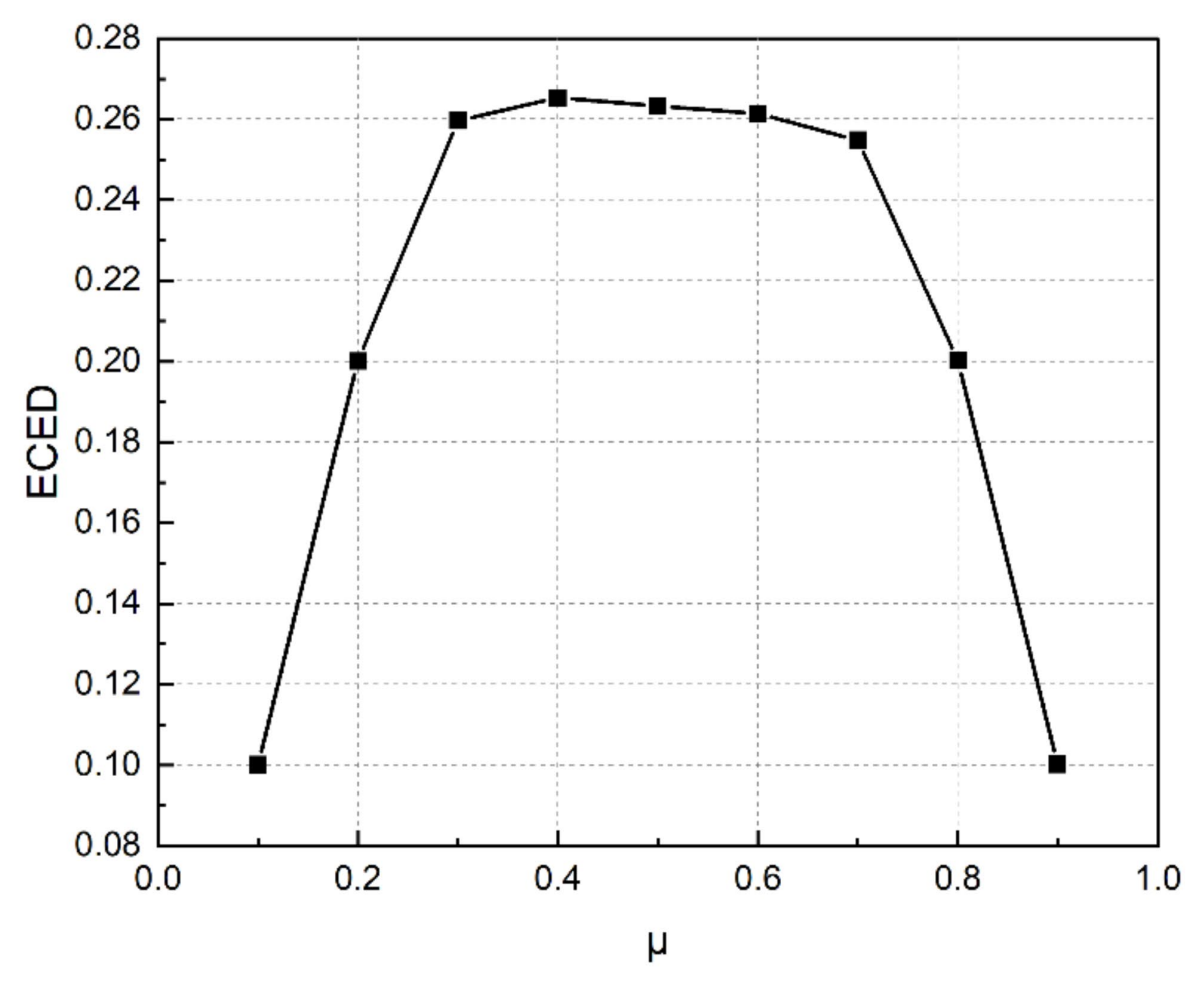
Change in values of WEED for change in µ.

**Fig. 14:**
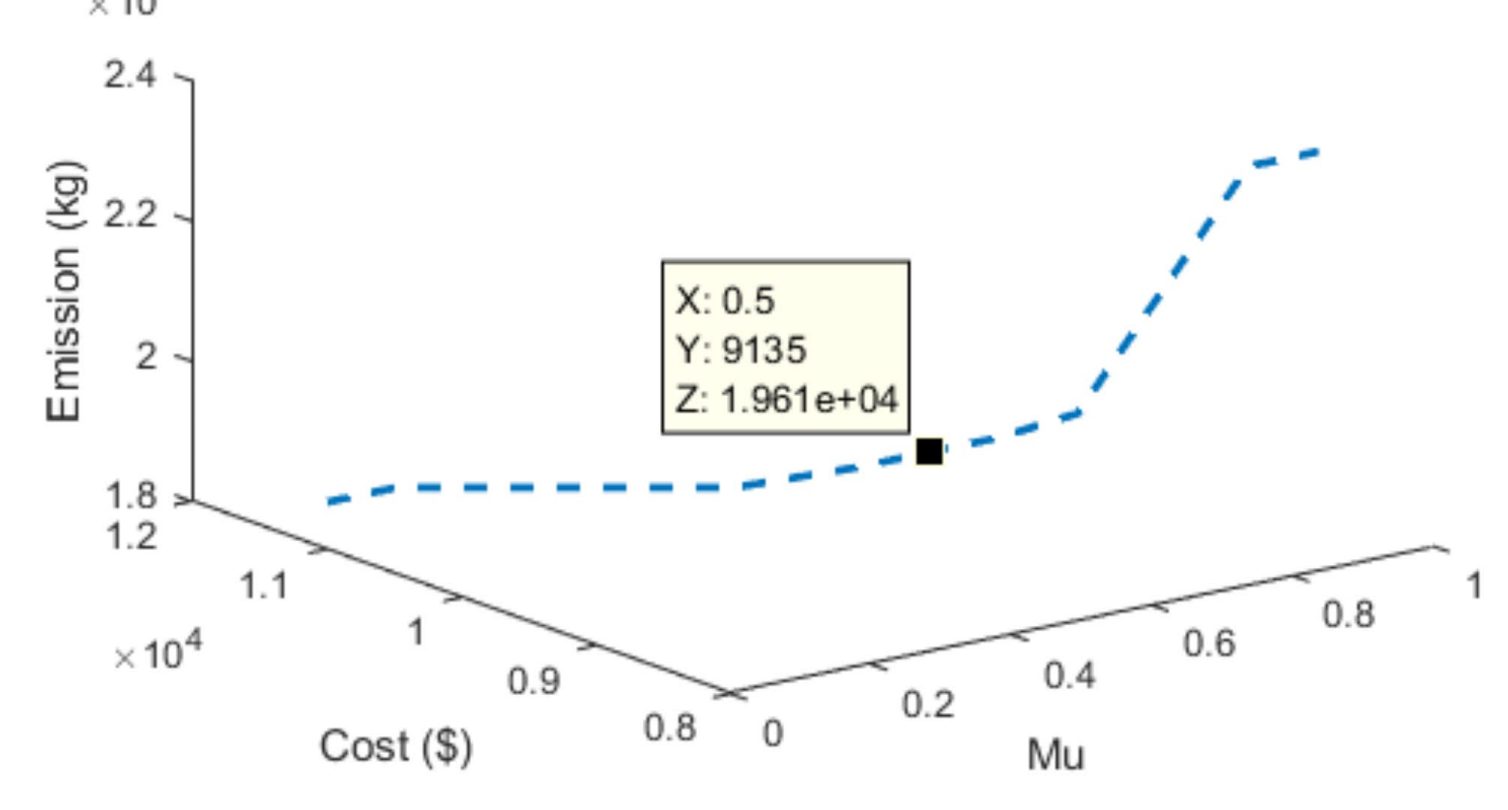
Generation cost and emission values for different values of µ (without DR).

Thereafter, WEED represented by Eq. (6) was minimized for values of µ varying from 0.1 to 0.9 and the cost of generation and emission was noted down. This was repeated for the load models without DR, with 30% and 40% DR. The change in the values of WEED (Eq. 6) for different values of µ can be seen in Fig. 13. The best compromised resolution between emission and cost of generation was obtained for µ = 0.5 for all load demand models and this is tabulated in Table 6. Figure 14 depicts the 3D model of generation cost and emission for change in the values of µ. The better compromised solution was pinned down in the figure for clarification.

## Conclusive remarks and future course of action

Six different scenarios are investigated in this paper, each of which are intended to minimize the cost of generation of the low voltage grid-connected microgrid system using a popular DE algorithm. An economic strategy called IBDR is also incorporated to further diminish the emission and cost limits of the subject microgrid system. The outcomes are directed at the following conclusion:

1. *IBDR* played a noteworthy role by reducing both the cost of generation and the emissions of the microgrid system. In addition, the results obtained show that they enhance the load factor of the system and decrease the peak demand.
2. *Grid Participation*: The cost of generation is minimum when the grid aggressively sells and buys power to and from the microgrid structure. Passive participation of the grid may perform as back up to cater to the power deficiency but does not contribute to minimizing the microgrid cost.
3. *Price in the electricity market*: The least cost of generation is obtained when the grid sells and buys power with the same TOU based electricity market pricing strategy. It has been observed in scenarios 4, 5 and 6 when the selling and buying price of power differs, the cost of generation is more than the ideal scenario (Scenario 1).
4. *A comparison with recently published literature* also corroborates to the fact that the total cost of the microgrid system which involves both the generation cost and incentives to be awarded to the customers is less than that of load shifting policy-based DSM wherein the incentives are not considered to benefit the customers.
5. *WEED* yields a well-adjusted compromised resolution between the emission and cost of generation which remained unattended when economic dispatch was emphasized alone.

### Limitations of the work

The presence of a battery energy storage system (BESS) or a PHEV charging station is a necessity to update the work in this research background which is missing in this work. This would also challenge the optimization algorithm to fulfill the complex constraints arising from the BESS and PHEV. Additionally, a hybrid of load shifting and curtailing policy can also be implemented on the load demand model and tested to study the efficiency and cost-effectiveness of the approach. Some other economic policies such as DSM and price-based demand response can be implemented for the same system and a comparison can be made for the same.

## Data Availability

Data will be made available on request to the corresponding author.

## Abbreviations

ELD: Economic load dispatch
ScELD: Static economic load dispatch
DcELD: Dynamic economic load dispatch
DSM: Demand side management
GA: Genetic algorithm
DE: Differential algorithm
DER: Distributed energy resource
PSO: Particle Swarm Optimization
CEED: Combined economic emission dispatch
DR: Demand response
DSM: Demand side management
CSA: Crow Search Algorithm
GWO: Grey Wolf Optimiser
CSAJAYA: Crow Search Algorithm and JAYA
ESS: Energy storage systems
EV: Electric vehicles
DRP: Demand response program
IBDR: Incentive based demand response
WEED: Weighted economic emission dispatch
MT: Micro turbine
WT: Wind turbine
NGFC: Natural gas fuel cell
CHP: Combined heat and power
TOU: Time of use
DE: Differential evolution
PHEV: Plug in hybrid electric vehicle
CFI: Carbon footprint index
